# Counteraction of Oxidative Stress by Vitamin E Affects Epigenetic Regulation by Increasing Global Methylation and Gene Expression of *MLH1* and *DNMT1* Dose Dependently in Caco-2 Cells

**DOI:** 10.1155/2018/3734250

**Published:** 2018-03-22

**Authors:** Katja Zappe, Angelika Pointner, Olivier J. Switzeny, Ulrich Magnet, Elena Tomeva, Jutta Heller, George Mare, Karl-Heinz Wagner, Siegfried Knasmueller, Alexander G. Haslberger

**Affiliations:** ^1^Department of Nutritional Sciences, University of Vienna, 1090 Vienna, Austria; ^2^Department of Toxicology, University Medical Center of the Johannes Gutenberg University Mainz, 55131 Mainz, Germany; ^3^Institute of Cancer Research, Department of Medicine I, Medical University of Vienna, 1090 Vienna, Austria

## Abstract

Obesity- or diabetes-induced oxidative stress is discussed as a major risk factor for DNA damage. Vitamin E and many polyphenols exhibit antioxidative activities with consequences on epigenetic regulation of inflammation and DNA repair. The present study investigated the counteraction of oxidative stress by vitamin E in the colorectal cancer cell line Caco-2 under normal (1 g/l) and high (4.5 g/l) glucose cell culture condition. Malondialdehyde (MDA) as a surrogate marker of lipid peroxidation and reactive oxygen species (ROS) was analyzed. Gene expression and promoter methylation of the DNA repair gene *MutL homolog 1* (*MLH1*) and the *DNA methyltransferase 1* (*DNMT1*) as well as global methylation by *LINE-1* were investigated. Results revealed a dose-dependent counteracting effect of vitamin E on H_2_O_2_-induced oxidative stress. Thereby, 10 *μ*M vitamin E proved to be more efficient than did 50 *μ*M in reducing MDA. Further, an induction of *MLH1* and *DNMT1* gene expression was noticed, accompanied by an increase in global methylation. Whether *LINE-1* hypomethylation is a cause or effect of oxidative stress is still unclear. In conclusion, supplementation of exogenous antioxidants like vitamin E *in vitro* exhibits beneficial effects concerning oxidative stress as well as epigenetic regulation involved in DNA repair.

## 1. Introduction

Lifestyle-associated diseases, such as cancer and cardiovascular, respiratory, and metabolic diseases, comprise most of the noncommunicable diseases and account for more than two-thirds of the worldwide deaths [1]. Natural bioactive nutritional compounds like vitamin E play a major role in nutrition-based disease improvements as well as in its prevention [2].

Vitamin E is a collective term including *α*, *β*, *γ*, and *δ* isomers of saturated tocopherols [3] and unsaturated tocotrienols [4]. Beneficial and harmful effects on human health by vitamin E were observed, and therefore usefulness of vitamin E is highly controversial. Intervention studies showed anti-inflammatory effects, a delay of the aging process [5, 6], anticancer properties [7, 8], antidiabetic and eye disease protective potential [9], and cardiovascular protective [10] features. Adjuvant vitamin E treatment of patients suffering from different cancer types led to controversial effects [11, 12]. A meta-analysis revealed an increased all-cause mortality by a high dose of vitamin E [13], while other studies found promising synergistic effects between vitamin E and administered drugs, especially anticancerous effects [14, 15]. Most studies are based on different isoforms of vitamin E or mixture ratios, or synthetic racemic or natural *R*-, *E*-configurated isomers, all leading to different biological effective doses.

One important mechanism of all vitamin E forms is the nonenzymatic antioxidative, radical scavenging potential by donating hydrogen from the phenolic group on the chromanol ring [16]. Reactive oxygen species (ROS), a group of reactive metabolic by-products affecting the redox balance, are essential for signaling pathways, detoxification, and host defense [17, 18]. Furthermore, they are known for modulating gene expression and regulating growth signals and therefore having a significant impact on the sustained proliferation of cancer. High levels of ROS occurring as a response to oxidative stressors such as exogenous agents including tobacco smoke, alcohol consumption, and infections or various inflammatory processes damage DNA, lipids, and proteins were found to upregulate oncogenes. Inflammation processes and aging per se are fueled by ROS [17, 18].

The major initial endogenous ROS is superoxide (O_2_^∙−^), which is generated from oxygen under NADH consumption or by NADPH oxidases (NOX) and xanthine oxidase (XO) [19]. It reacts spontaneously with nitric oxide (NO^∙^) to peroxynitrite (ONOO^−^) or is disproportionated by superoxide dismutase (SOD) to hydrogen peroxide (H_2_O_2_) [19]. *In vitro* studies showed that exogenous H_2_O_2_ is linked with increased cell proliferation attended by moderate ROS concentrations [20]. Downstream ROS deriving as singlet oxygen _ _^1^O_2_ are able to oxidize aliphatic chains to fatty acids, which are substrates for the hydroxyl radical to generate fatty acid peroxide radicals [21]. Peroxidation of polyunsaturated fatty acids (PUFAs) leads to the formation of small aldehydes such as malondialdehyde (MDA) and trans-4-hydroxy-2-nonenal (4-HNE) [22]. Both are considerably involved in cellular signaling, affecting chromatin modifications [23].

Long-term oxidative stress leads to chronic changes in enzymatic, transcriptional, epigenetic, and genomic regulation by inducing a new steady-state level of oxidants and antioxidants [18]. In cancer development, ROS-generating processes are generally upregulated and promote cell proliferation by altering metabolism and cell control mechanisms, consequently sustaining DNA damage, genomic instability, and inflammation [24].

Emerging studies reveal that only 5–10% of cancer incidences are exclusively caused by genetic factors [25]. In most other cases, epigenetic alterations play an important part [26]. Linkages of epigenetics with oxidative stress, nutritional effects, and cell signaling underline its importance [27]. Among epigenetic mechanisms, DNA methylation at the 5-position of cytosines in cytosine-guanine sequences (CpGs) in promoter regions is a key control mechanism of gene expression [28]. A global surrogate marker for estimating the genomic DNA methylation constitutes mobile element *long interspersed nuclear element-1* (*LINE-1*) with a frequency of 17% of the human genome and an estimated total genomic methylation content of 1/3 [29, 30]. Genome-wide loss of DNA methylation leads to genomic instability and results in a higher chance of mitotic recombination [31]. Therefore, *LINE-1* is suggested as an indicator of genomic stability [32]. The key enzymes involved in DNA methylation constitute the family of DNA methyltransferases (DNMTs) mediating the transfer of methyl groups to cytosines. DNMT overexpression or activation participates in tumor suppressor silencing [33, 34]. Tumors also often show aberrant high promoter methylation of the mismatch repair (MMR) gene *MutL homolog 1* (*MLH1*), which further boosts genomic instability [35]. Moreover, synergistic oxidative DNA damage repair plays a crucial role to protect the genome and to ensure its stability. It further reveals the complex interplay of the individual repair systems [36]. MMR was reported to contribute to base excision repair (BER) of 8-oxo-2′-deoxyguanosine (8-oxo-dG) [37] as well as to nucleotide excision repair (NER) of the MDA adduct with deoxyguanosine (M1dG) [38, 39]. MMR proteins, especially MLH1, were found to interact with DNMTs, the NAD-dependent deacetylase sirtuin-1 (SIRT1) and poly(ADP-ribose) polymerase 1 (PARP1) to prevent altered gene transcripts from the damaged site and induce cell death, when damage exceeds the repair capacity [40, 41].

The focus of this study was to investigate epigenetic effects of vitamin E in counteracting H_2_O_2_-induced ROS production and lipid peroxidation. Therefore, colorectal adenocarcinoma Caco-2 cells in normoglycemic and hyperglycemic media were treated with a mixture of tocopherols and tocotrienols in combination with different doses of H_2_O_2_ as well as ROS inhibitor *N*-acetylcysteine (NAC) [42] and NOX inhibitor VAS2870 [43]. Our aim was to identify concentrations of H_2_O_2_ and vitamin E, which are worth further investigation in higher repetition. Thus, total ROS, superoxide level, and MDA levels were assessed. To evaluate epigenetic alterations in genes linked with oxidative stress, chromosomal integrity, and DNA repair, global methylation by *LINE-1* promoter methylation as well as promoter methylation and expression of *MLH1* and *DNMT1* were determined.

## 2. Material and Methods

### 2.1. Cell Culture

The adherent human colorectal adenocarcinoma cell line Caco-2 (DSMZ, Germany) was cultured as monolayer in Dulbecco's modified Eagle medium (DMEM) high glucose (4.5 g/l) supplemented with 0.584 g/l l-glutamine, 5% (w/v) penicillin/streptomycin, and 10% (v/v) FBS at 37°C in a humidified atmosphere of 95% air and 5% CO_2_. Cells were passaged before reaching confluency, using 1x PBS and Accutase® solution. A fraction of these cells was adjusted by stepwise reduction of d-glucose through addition of DMEM normal glucose (1.0 g/l, supplemented like the high glucose DMEM) to, finally, 1.0 g/l d-glucose. In the first step, d-glucose concentration was reduced by 1.75 g/l and cultivated for 14 days. The second and third reductions were by 0.875 g/l with cultivation for 8 and at least 23 days, respectively. Cultivation after each reduction was performed to give the cells time to adapt to the new condition, especially before the analyses (all chemicals from Sigma-Aldrich, Vienna).

### 2.2. Cell Treatments

Cells were seeded in 6-well plates in media with respective glucose concentration. The untreated control was incubated 72 h to reach 90% confluency. For treatment, after 24 h of growth, cells were treated for 48 h with 0, 25, 50, 250, or 500 *μ*M H_2_O_2_ (Sigma-Aldrich, Vienna) and cotreated with 0, 10, or 50 *μ*M vitamin E (Aqua-E® supplement YASOO Health Inc., Nicosia) in all combinations. Vitamin E comprised a mixture of micellized *d*-*α*-tocopherol 20 IU/ml, other tocopherols 15 mg/ml, and tocotrienols 2 mg/ml from natural origin. Media without phenol red were used to avoid interference in treatments for ROS/superoxide and MDA detection. To investigate the contribution of ROS as a potential modulator of epigenetic alterations, cells were treated with 1 mM NAC (Enzo Life Science, Lausen) dissolved in sterile deionized water (QIAGEN, Hilden) or 2 *μ*M VAS2870 (Sigma-Aldrich, Vienna) dissolved in DMSO (Sigma-Aldrich, Vienna). Further, after 1 h pretreatment with either NAC or VAS2870, 250 *μ*M H_2_O_2_ was applied. Controls were treated with the corresponding solvent only. For analyzing the impact of the treatments, cells were harvested using 1x PBS and Accutase solution.

### 2.3. MDA as Marker for Lipid Peroxidation

Harvested cells were counted, and MDA levels were determined via HPLC with fluorescent detection at 533 nm as previously described [44, 45]. All chemicals were purchased from Sigma-Aldrich, Vienna, and all organic solvents used were of HPLC grade and purchased from Rathburn Chemicals Ltd., Walkerburn. Resulting MDA levels were expressed in MDA concentration per cell number. For run comparison, MDA levels were related to high glucose untreated control and corresponding media control for analysis of treatment impact.

### 2.4. Total ROS and Superoxide Level

1 × 10^5^ treated cells per well of a 96-well plate with black walls and transparent flat bottom were seeded by centrifugation at 40 ×g, 3 min with lowest acceleration (acc. 1) and second lowest deceleration (dcl. 2) (Jouan BR4i Multifunction Centrifuge, Thermo Fisher Scientific). All other steps were performed according to ROS/superoxide detection kit ENZ-51010 (Enzo Life Science, Lausen) manufacturers' instructions except after adding 1x wash buffer, an additional centrifugation step (same conditions as described above) was conducted to bring loosened cells down to the bottom. For the 60 min staining, a 1 : 2500 dilution of each dye in respective (glucose) DMEM was used. The negative assay control was generated by ROS scavenging activity for 60 min with 5 mM NAC and the positive assay control by ROS induction with 400 *μ*M pyocyanin for 20 min of untreated cells. Plates were read at 37°C with FLUOstar Optima microplate reader (BMG Labtech, Ortenberg) using 4 mm orbital averaging, 6 cycles with 10 flashes per well, and cycle and fluorescence filters with ex 485 nm/em 520 nm and ex 544 nm/em 612 nm. Fluorescence levels were calculated over the same measurement time for all plates. Corresponding ROS/superoxide levels were related to high glucose untreated control for run comparisons and corresponding media control for analysis of treatment impact.

### 2.5. RNA/gDNA Extraction and Bisulfite Conversion

RNA and gDNA were extracted simultaneously from treated cells using RNAprotect® Cell Reagent, AllPrep DNA/RNA Mini Kit with additional reagent DX for lysis and proteinase K, and RNase-free DNase for cleanup (all QIAGEN, Hilden) according to manufacturer's protocols for cell culture. Homogenization step was performed using stainless steel beads (QIAGEN, Hilden) with Precellys® 24 (Bertin Technologies, Montigny-le-Bretonneux) at 1600 ×g (5000 rpm), 2x 15 s with a 10 s break in between. Bisulfite conversion of unmethylated cytosines in DNA was performed according to EpiTect® Fast Bisulfite Conversion Kit (QIAGEN, Hilden) manufacturer's instructions using bisulfite reaction setup for high-concentration samples and extension of both 60°C incubation times to 20 min. RNA and DNA concentrations were determined with Pico100 UV/Vis spectrophotometer (Picodrop Limited, Hinxton).

### 2.6. Gene Expression Analysis

Reverse transcription and cDNA amplification were done either with 1 *μ*g RNA using the RT^2^ First Strand Kit (QIAGEN, Hilden) or with the following modifications for the RT^2^ HT First Strand Kit for 96 samples (QIAGEN, Hilden). GE2 buffer and RT mix were immediately aliquoted and stored for later use at −20°C. 8 *μ*l of RNA solution was mixed with 6 *μ*l GE2 buffer in a PCR tube and incubated in the thermocycler with preheated lid for 15 min at 42°C and then 5 min at 95°C. 6 *μ*l RT mix was added and incubated in the thermocycler with preheated lid for 5 min at 37°C. After mixing with 91 *μ*l nuclease-free distilled water, the cDNA was stored at −20°C.

Real-Time PCR was performed using *GAPDH* as housekeeping gene and *DNMT1* and *MLH1* as genes of interest according to RT^2^ qPCR Primer Assays and RT^2^ SYBR Green ROX qPCR Mastermix manufacturer's protocol (all QIAGEN, Hilden) in the real-time thermocycler StepOnePlus™ (Applied Biosystems, Vienna). PCR conditions were an initial PCR activation step of 10 min at 95°C and a 40x repeated 2-step cycling of 15 s denaturation at 95°C, and 1 min annealing and extension at 60°C followed by a melt curve analysis from 60°C to 95°C in 0.3°C steps. For comparisons of runs, an untreated control of each culture media was used on every plate. Relative expression was calculated using ΔΔC_T_ method and was expressed as 2^−ΔΔC_T_^.

### 2.7. Standard Synthesis for Methylation-Sensitive High-Resolution Melting (MS-HRM)

For synthesis of unmethylated DNA standards, purified gDNA from untreated Caco-2 (high glucose media) was amplified according to the REPLI-g® Mini Kit (QIAGEN, Hilden) handbook using 5 *μ*l template DNA and 16 h incubation with Master Mix at 30°C. Amplified gDNA was purified by precipitation with sodium acetate according to QIAGEN FAQ ID-305. Therefore, 1/10 volume of 3 M sodium acetate (Sigma-Aldrich, Vienna), pH 5.2, and 2–2.5 volumes ice-cold 96% ethanol (analysis grade, VWR Chemicals, Vienna) were mixed with the gDNA and precipitated for 1 h at −20°C. After centrifugation at 4°C for 20 min with 21200 ×g, the alcohol was pipetted off, and the pellet was washed twice at room temperature with 70% ethanol and was air dried. The purified DNA was dissolved according to pellet size in about 50–100 *μ*l sterile TE buffer (AppliChem, Darmstadt). A part of the unmethylated standard was methylated according to the manufacturer's protocol for CpG Methyltransferase M.SssI 20 U/*μ*l using 640 *μ*M *S*-adenosyl-l-methionine (SAM) and 10x NEBuffer 2 (all NEB, Frankfurt). Methylation at 37°C was performed for 4 h with addition of fresh SAM in between. The methylated standard was purified with sodium acetate as the unmethylated one. Unmethylated cytosines in standard DNA were bisulfite converted according to EpiTect Bisulfite Kit handbook (QIAGEN, Hilden) using 0.63–2 *μ*g DNA.

### 2.8. DNA Methylation Analysis by MS-HRM

MS-HRM was performed according to the EpiTect HRM™ PCR handbook (QIAGEN, Hilden) using the Rotor-Gene® Q (QIAGEN, Hilden). The 10 *μ*l reaction mix for PCR contained 5 *μ*l 2x EpiTect HRM PCR Master Mix (QIAGEN, Hilden), 5 ng bisulfite converted DNA, and RNase-free water (QIAGEN, Hilden). Further PCR conditions were optimized for every primer set (http://biomers.net GmbH, Ulm). For *MLH1*, 500 nM of each primer (Table 1) was used. Initial PCR activation step 5 min at 95°C was followed by 40x repeated 3-step cycling of 15 s denaturation at 95°C, 30 s annealing at 50°C, and 20 s extension at 72°C. After 1 min denaturation at 95°C and 1 min heteroduplex formation at 40°C, HRM analysis was performed from 55°C to 95°C in 0.2°C steps. For *DNMT1*, 750 nM of each primer (Table 1) and 0.29 mM additional MgCl_2_ were used. Initial PCR activation step 5 min at 95°C was followed by 40x repeated 3-step cycling of 15 s denaturation at 95°C, 30 s annealing at 50°C, and 20 s extension at 72°C. After 1 min at 95°C and 1 min at 40°C, HRM analysis was performed from 55°C to 95°C in 0.2°C steps. For *LINE-1*, 750 nM of each primer was used (Table 1). The initial hot-start polymerase activation step 5 min at 95°C was followed by 45x repeated 3-step cycling of 30 s denaturation at 95°C, 45 s annealing at 54°C, and 30 s extension at 72°C. After 1 min at 95°C and 1 min at 45°C, HRM analysis was performed from 60°C to 95°C in 0.1°C steps. DNA methylation standards were checked for every primer pair for either complete demethylation or methylation by comparison to EpiTect PCR Control DNA set standards (QIAGEN, Hilden). For comparison of all MS-HRM runs, unmethylated and methylated standards were mixed and used from the same mixture aliquots to generate the calibration curves at each run.

MS-HRM data were analyzed with Rotor-Gene Q Series Software version 2.3.1 according to Rotor-Gene Q User Manual (QIAGEN, Hilden). Normalized curves were analyzed based on curve interpolation as published by Spitzwieser et al. [46] based on Migheli et al. [47] and via the standardized fluorescence (SF) for the maximal temperature span in which the curves still differ. Calibration curve fitting was performed using TableCurve 2D and SigmaPlot (both Systat Software Inc.) for identifying the most suitable simple equation for each gene.

### 2.9. Statistical Analysis

Data are represented as mean ± standard deviation (SD). All experiments were performed in duplicates, if not indicated otherwise. Data presentation and statistical analysis were performed with SPSS® Statistics version 23 (IBM). Two-tailed Student's *t*-test for independent samples (CI = 0.95) without Welch correction was used for comparison with the untreated control (c) or respective H_2_O_2_ treatment (h). Two-way ANOVA was used for comparison of the different treatments (t) in the context of different glucose concentrations (g). Pearson correlation was performed between the measured parameters. Differences were considered statistically significant at a *p* value ≤ 0.05.

## 3. Results

### 3.1. Effects of Glucose Concentration

**Figure 1:**
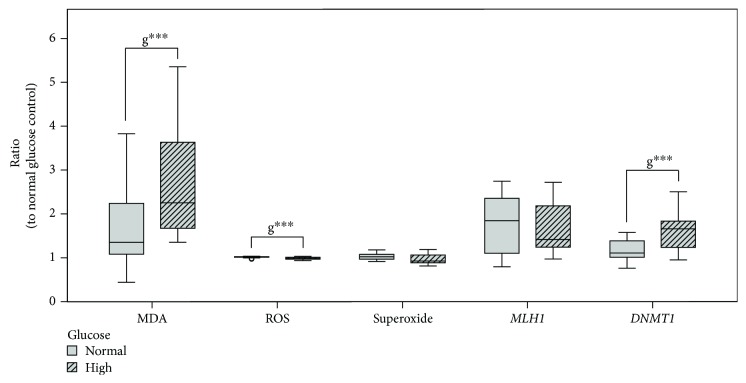
Impact of glucose level and shared 48 h treatments (Table 2) in Caco-2 on lipid peroxidation (MDA level) *n* = 52, ROS *n* = 39, and superoxide *n* = 39 formation and on *MLH1n* = 36 and *DNMT1n* = 36 gene expression. Data are displayed as ratio to normal glucose untreated control and grouped by normal and high glucose growing conditions. Differences between groups are statistically analyzed by two-way ANOVA on ratios except for gene expression, where analysis is based on ΔΔC_T_ to normal glucose untreated control. Significance by glucose (g) is marked with ∗∗∗ for *p* ≤ 0.001.

Cells were grown in normoglycemic media (1 g/l) as well as hyperglycemic conditions (4.5 g/l d-glucose), reflecting severe diabetic blood level [48], to elucidate the influence of the glucose concentration. Glucose concentration significantly (*p* ≤ 0.01) increased MDA levels in cells grown under hyperglycemic compared to those in normoglycemic conditions (Figure 1). ROS levels were significantly decreased in relation to normoglycemic untreated control (*p* ≤ 0.001). However, superoxide level was not affected by the hyperglycemic condition.

**Figure 2:**
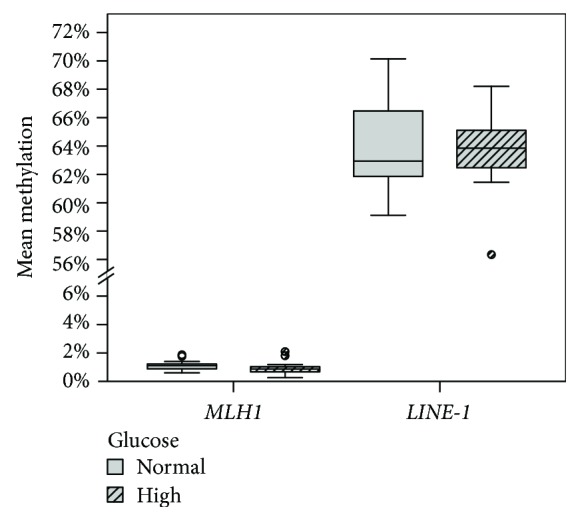
Impact of glucose levels and shared 48 h treatments (Table 2) in Caco-2 cells on the mean *MLH1* promoter *n* = 36 and global methylation (*LINE-1* promoter region) *n* = 36 grouped by normal and high glucose conditions. Differences between groups were statistically analyzed on mean promoter methylation by two-way ANOVA. No significance by glucose (g) was found.

*MLH1* expression as well as its mean promotor methylation was not affected by the glucose concentration alone (Figures 1 and 2). *DNMT1* expression was significantly increased by glucose in treated cells (*p* ≤ 0.001) (Figure 1). *LINE-1* promoter methylation was not affected by different glucose concentrations (Figure 2).

Consequently, for analysis of the treatment effects in the following sections, relative comparison to respective untreated glucose control was used to minimize the effects of the glucose level. Furthermore, it would be of interest to investigate these observed changes by glucose reduction. Further investigation by measuring ROS, MDA levels, and *DNMT1* expression during smaller reductions steps based on these findings might reveal the mechanisms behind in more detail.

### 3.2. Effects of Vitamin E on Oxidative Stress

#### 3.2.1. MDA

**Figure 3:**
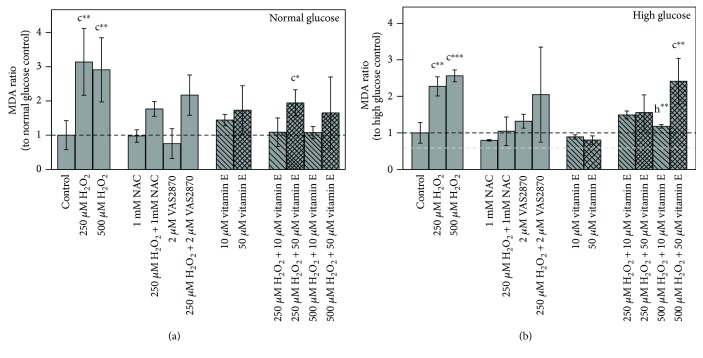
Impact of 48 h treatments on lipid peroxidation in Caco-2 cells grown in (a) normal and (b) high glucose media. Bar charts display the mean ± SD of MDA ratio to respective glucose untreated control. The gray dashed line represents the level of normal glucose untreated control in relation to high glucose untreated control. Differences to the respective controls are statistically analyzed by Student's *t*-test. Significance to untreated control (c) and respective H_2_O_2_ treatment (h) is marked with ∗ for *p* ≤ 0.05, ∗∗ for *p* ≤ 0.01, and ∗∗∗ for *p* ≤ 0.001. Untreated controls are *n* = 5 due to assay controls.

Incubation with 250 *μ*M as well as 500 *μ*M H_2_O_2_ provoked a significant increase in MDA levels at both glycemic conditions (*p* ≤ 0.01) (Figure 3). Addition of 10 *μ*M or 50 *μ*M vitamin E could alleviate this effect. Interestingly, a dose of 10 *μ*M vitamin E was more effective and completely prevents H_2_O_2_-induced MDA. In the normoglycemic condition, significant higher MDA levels compared to those in the untreated controls were measured after incubation with 250 *μ*M H_2_O_2_ + 50 *μ*M vitamin E (*p* ≤ 0.05), whereas both vitamin E concentrations at high glucose levels in combination with 250 *μ*M H_2_O_2_ led to the equal MDA levels.

The ROS scavenger NAC as well as the NOX inhibitor VAS2870 was able to reduce the MDA levels following 250 *μ*M H_2_O_2_ treatments. A single treatment with NAC or VAS2870 had no significant effect on the MDA level.

Treatment with 10 *μ*M or 50 *μ*M vitamin E alone led to increased MDA levels under normoglycemic conditions (Figure 3(a)). Under hyperglycemic conditions, 10 *μ*M or 50 *μ*M vitamin E alone led to reduced MDA levels (Figure 3(b)). However, the latter was not significant.

#### 3.2.2. ROS/Superoxide

**Figure 4:**
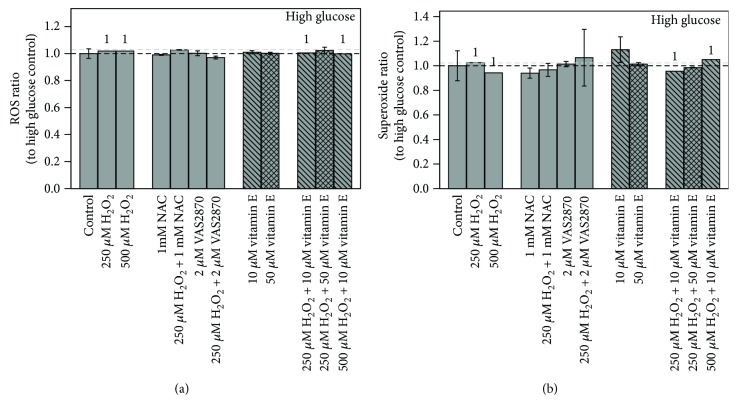
Impact of 48 h treatments on (a) ROS and (b) superoxide levels in high glucose grown Caco-2 cells. Bar charts display the mean ratio ± SD to high glucose untreated controls. The gray dashed line represents the level of normal glucose untreated control in relation to high glucose untreated control. Differences to respective controls are statistically analyzed by Student's *t*-test. 1 indicates lacking replicate, *n* = 1. Untreated controls are *n* = 4 due to assay controls.

Treatment with the assay controls pyocyanin or NAC resulted in the expected ROS/superoxide alterations. 48 h incubation with H_2_O_2_, vitamin E, or ROS/NOX inhibitors had no significant impact on ROS or superoxide levels at both glycemic conditions (Figure 4), indicating that no stable changes were generated.

### 3.3. Treatment on *MLH1* and *DNMT1* Gene Regulation and *LINE-1* Methylation

#### 3.3.1. *MLH1* Expression

**Figure 5:**
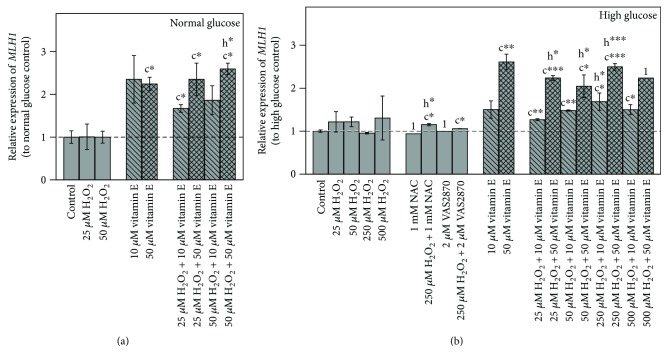
Impact of 48 h treatments on *MLH1* gene expression in (a) normal and (b) high glucose grown Caco-2 cells. Bar charts display the mean ± SD to respective glucose untreated control. The gray dashed line represents the level of normal glucose untreated control in relation to high glucose untreated control. Differences to respective controls are statistically analyzed on ΔΔC_T_ to normal glucose untreated control by Student's *t*-test. Significance to untreated control (c) and respective H_2_O_2_ treatment (h) is marked with ∗ for *p* ≤ 0.05, ∗∗ for *p* ≤ 0.01, and ∗∗∗ for *p* ≤ 0.001. 1 indicates lacking replicate, *n* = 1.

There was no significant effect on *MLH1* expression after an exclusive treatment with H_2_O_2_ at various concentrations under both glycemic conditions (Figure 5). Incubation with solely 10 *μ*M (not significant) or 50 *μ*M (*p* ≤ 0.05) vitamin E resulted in an elevated expression of *MLH1*.

Furthermore, a combined treatment of vitamin E and H_2_O_2_ in normoglycemic media significantly increased *MLH1*. A similar enhancing effect could be observed under hyperglycemic conditions, where combinations of 10 or 50 *μ*M vitamin E with 25 to 500 *μ*M H_2_O_2_ resulted in a significantly higher expression of *MLH1* (*p* ≤ 0.05). Concerning treatment with 500 H_2_O_2_ + 50 *μ*M vitamin E only, data from one sample could be obtained as this concentration was highly cytotoxic.

Inhibitor studies with NAC or VAS2870 combined with H_2_O_2_ performed in the hyperglycemic condition both showed a significant increase of *MLH1* expression.

#### 3.3.2. *DNMT1* Expression

**Figure 6:**
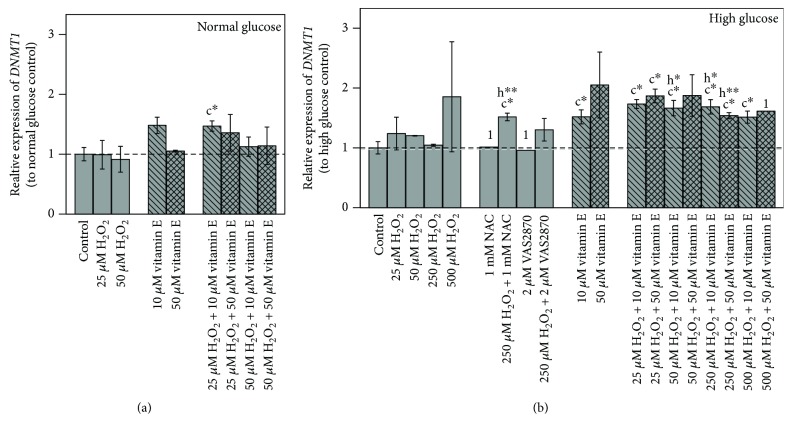
Impact of 48 h treatments on *DNMT1* gene expression in (a) normal and (b) high glucose grown Caco-2 cells. Bar charts display the mean ± SD to respective glucose untreated control. The gray dashed line represents the level of normal glucose untreated control in relation to high glucose untreated control. Differences to respective controls are statistically analyzed on ΔΔC_T_ to normal glucose untreated control by Student's *t*-test. Significance to untreated control (c) and respective H_2_O_2_ treatment (h) is marked with ∗ for *p* ≤ 0.05 and ∗∗ for *p* ≤ 0.01. 1 indicates lacking replicate, *n* = 1.

In cells grown under normoglycemic conditions, 10 *μ*M vitamin E increased *DNMT1* expression, while 50 *μ*M showed no effect (Figure 6(a)). At hyperglycemic conditions, both vitamin E concentrations increased *DNMT1* expression, which was significant for 10 *μ*M (*p* ≤ 0.05) (Figure 6(b)). In contrast, H_2_O_2_ alone scarcely affected *DNMT1* expression level.

Combined incubation of vitamin E and H_2_O_2_ increased *DNMT1* expression under both glycemic conditions and was significant under hyperglycemic conditions (*p* ≤ 0.05). Thereby, high H_2_O_2_ concentrations (250 *μ*M, 500 *μ*M) increased *DNMT1* expression less than did moderate ones (25 *μ*M, 50 *μ*M).

A significant increase to the corresponding H_2_O_2_ treatment control was also detected for combinations of 10 *μ*M vitamin E with 50 *μ*M and 250 *μ*M H_2_O_2_ (*p* ≤ 0.05) and for 250 *μ*M H_2_O_2_ + 50 *μ*M vitamin E (*p* ≤ 0.01).

NAC and VAS2870, when incubated with 250 *μ*M H_2_O_2_, both showed a trend towards an upregulation compared to untreated and H_2_O_2_ treatment controls, which was more pronounced and significant with NAC (*p* ≤ 0.05).

#### 3.3.3. Promoter Methylation of *MLH1*, *DNMT1*, and *LINE-1*

**Figure 7:**
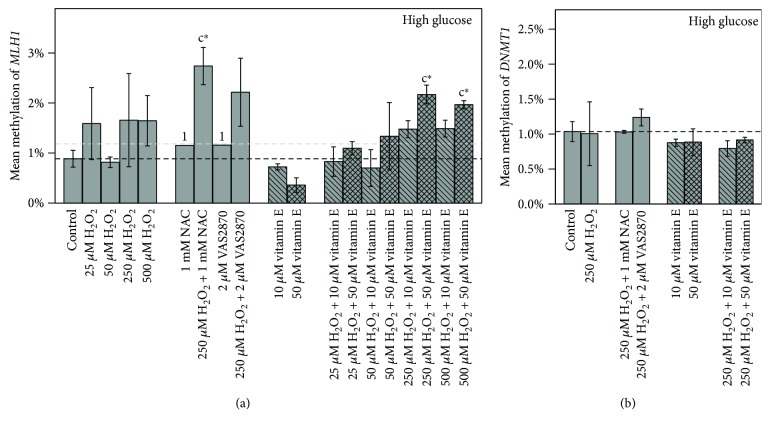
Impact of 48 h treatments with vitamin E on (a) *MLH1* and (b) *DNMT1* mean promoter methylation in high glucose grown Caco-2 cells. Bar charts display the mean ratio ± SD to high glucose untreated control. The gray dashed line represents the level of normal glucose untreated control. Differences to respective controls are statistically analyzed by Student's *t*-test. Significance to untreated control (c) is marked with ∗ for *p* ≤ 0.05. 1 indicates lacking replicate, *n* = 1.

Promoters of *MLH1* and *DNMT1* were both unmethylated in all treatments within a range of 0.4%–2.7% and 0.8%–1.2% methylation degree, respectively (Figure 7).

Consistently for both genes, no correlations between gene expression and methylation level were found. However, both gene expression levels themselves correlated positively (*r* = 0.488, *p* ≤ 0.01, *n* = 28). Furthermore, a positive correlation was observed for MDA level and *MLH1* methylation (*r* = 0.514, *p* ≤ 0.05, *n* = 16).

**Figure 8:**
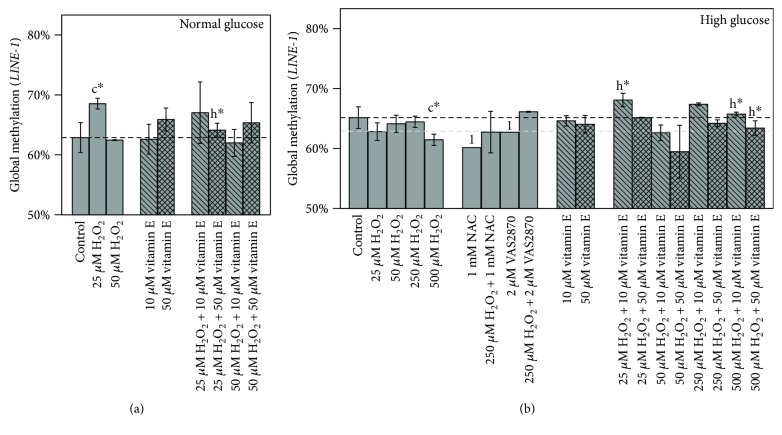
Impact of 48 h treatments on global methylation in (a) normal and (b) high glucose grown Caco-2 cells. Bar charts display the mean ± SD of *LINE-1* methylation levels. The gray dashed line represents the level of normal glucose untreated control in relation to high glucose untreated control. Differences to respective controls are statistically analyzed by Student's *t*-test. Significance to untreated control (c) and respective H_2_O_2_ treatment (h) is marked with ∗ for *p* ≤ 0.05.

Caco-2 featured higher genomic instability as seen by low *LINE-1* promoter methylation level in the normoglycemic (62.4%) and hyperglycemic (64.7%) untreated controls (Figure 8). Treatment of 25 *μ*M H_2_O_2_ under normoglycemic conditions led to a significant increase in *LINE-1* methylation (*p* ≤ 0.05), which was reduced by a combined treatment with 50 *μ*M vitamin E (*p* ≤ 0.05) (Figure 7(a)). Under hyperglycemic conditions, H_2_O_2_ tended to reduce *LINE-1* methylation with a concentration of 500 *μ*M causing a clear decrease (*p* ≤ 0.05). The combination of 25 *μ*M or 500 *μ*M H_2_O_2_ with 10 *μ*M vitamin E caused a significant increase in global methylation compared to corresponding H_2_O_2_ treatment (*p* ≤ 0.05).

Incubation with NAC or VAS2870 under hyperglycemic conditions showed a trend to further decrease *LINE-1* methylation. However, when combined with H_2_O_2_, *LINE-1* methylation was higher compared to respective treatments of inhibitors alone.

## 4. Discussion

Obesity is one of the leading causes of type 2 diabetes, clinically characterized by chronic hyperglycemia. Both medical conditions often entail numerous comorbidities including cancer, metabolic syndrome, and cardiovascular and neurodegenerative diseases, resulting in an increased mortality risk [49]. Obesity and diabetes have been consistently associated with higher levels of oxidative stress, with hyperglycemia as one primary discussed contributor. Accumulation of ROS is a central mediator of cellular damage and intracellular signaling pathways, playing a pivotal role in the progression of diabetes and development of complications [50, 51].

Plants can synthesize a wide range of nonenzymatic antioxidants such as polyphenols or vitamins to scavenge ROS. Supplementation of exogenous antioxidants constitutes a potential means to counteract ROS-induced oxidative damage [16, 17]. Despite already discussed possible adverse effects, vitamin E exhibits strong antioxidative activities and impacts multiple regulatory pathways with consequences on epigenetic regulation of genes involved in the processes of inflammation and DNA repair [18, 52].

### 4.1. Effect of Glucose Level

The comparison of Caco-2 cells grown under normoglycemic media or under the frequently used hyperglycemic cell culture media in this study elucidated potential effects of severe diabetic glucose blood level [48] and further revealed possible influence of glucose level on treatments with H_2_O_2_ and/or vitamin E. A previous study has already remarked on the effects of high glucose on multiple signaling pathways targeting cell growth and maintenance, cell cycle, and cell proliferation in human hepatocellular carcinoma cell lines [53]. In our study, we could observe glucose-induced elevated *DNMT1* expression as well as an increase in lipid peroxidation assessed by MDA level during treatments (Figure 1). In contrast to studies on rats and diabetic patients [54], in our study on Caco-2 cells, increased MDA levels were accompanied by a reduction of ROS. One possible explanation for this finding might be the Warburg effect, an altered metabolism in cancer cells. This mechanism is characterized by an increased demand of glucose by higher glycolysis and pentose-phosphate pathway (PPP) rate, resulting in increased NADPH levels, which in turn drive down ROS levels to prevent oxidative stress-induced cell death [24, 55].

### 4.2. Treatment Effects on Lipid Peroxidation

We could demonstrate that vitamin E was able to reduce H_2_O_2_-induced lipid peroxidation dose dependently in both glucose conditions with 10 *μ*M vitamin E being more potent than 50 *μ*M. The difference in effects between vitamin E concentrations could be explained by the prooxidative potential of vitamin E in higher doses as reported previously [13]. When applied alone under hyperglycemic conditions, vitamin E was able to decrease MDA levels. These were still higher than those of the normoglycemic untreated control. In contrast, under normoglycemic conditions, vitamin E slightly increased MDA levels. Assuming that normoglycemic conditions led to low absolute ROS levels, addition of vitamin E might cause a redox misbalance by an excess of antioxidants.

### 4.3. Treatment Effects on DNA Damage Repair

MDA is not merely a by-product of lipid peroxidation but is also responsible for signaling and forms DNA adducts, such as M1dG, which, if not repaired, becomes mutagenic [23]. Consequently, at higher MDA levels, an increased DNA repair, for example, via an upregulation of the repair gene *MLH1*, is of advantage and might be cancer protective [38, 39].

Our results showed that such an effect was not caused by increased oxidative stress induced by the H_2_O_2_ treatments, which did not alter *MLH1* expression despite the highest MDA levels. In contrast, vitamin E was rather effective in this regard, showing a significant induction of *MLH1* over all treatments. It would be of interest to assess oxidative DNA damage level in further studies, to elucidate if vitamin E-induced *MLH1* expression was solely concentration dependent or stimulated by increased DNA damage.

*MLH1* is reported to show aberrant high methylation patterns in so-called CIMP- (CpG island methylator phenotype-) positive tumors, first identified in colorectal cancer. [35]. CIMP-negative Caco-2 cells [56] displayed very low methylation rates (0.4%–2.7%) over all treatments, suggesting that regulation of *MLH1* expression in non-CIMP cancer types rather lies beyond DNA methylation. Consequently, our results showed no correlation between gene expression and methylation level of *MLH1* as previously also reported [57]. Moreover, *MLH1* promoter methylation was not affected by increased *DNMT1* expression. However, a positive correlation was observed for MDA level and *MLH1* methylation. This could indicate that high amounts of damage and induced ROS might provoke development towards CIMP.

### 4.4. Treatment Effects on *DNMT1*

We could demonstrate that H_2_O_2_ treatment combined with inhibitors as well as vitamin E led to an elevated *DNMT1* expression, though exclusive treatment with H_2_O_2_ scarcely affected *DNMT1* expression_._ These effects were more pronounced under hyperglycemic conditions, suggesting a significant glucose-induced impact.

Our results further showed a positive correlation between *DNMT1* and *MLH1* expression. Their proteins are reported to interact with each other [40, 41] and these genes to be controlled in a cell cycle-dependent manner with a gene expression restricted to S-phase for *DNMT1* [58] and an upregulated one for *MLH1* [59]. Furthermore, vitamin E compounds are known to be potent cell cycle modulators [15, 60].

These studies support our findings that the modulation of *DNMT1* and *MLH1* in Caco-2 by vitamin E might be based on a S-phase block. Cell cycle arrest with increased expression of DNA repair genes following DNA damage responses is thereby of particular advantage [61].

Consistently, the additional incubation with high H_2_O_2_ concentrations that enhanced *MLH1* expression more and *DNMT1* expression less than did moderate concentrations could be observed. ROS, triggering proliferation by the redox regulated cell cycle [24, 62], might promote S-phase transit despite the increased damage. Further experiments could clarify our assumptions on the cell cycle arrest in S-phase as causing a cell cycle arrest in the mutant p53 Caco-2 cells [63] by an independent mechanism provides a potential treatment to combat p53-defective cancer types.

### 4.5. Treatment Effects on *LINE-1*

DNA methylation of repetitive elements such as *LINE-1* can serve as a surrogate marker for global genomic DNA methylation, as it occurs with a frequency of at least 17% [29] in the human genome. Global DNA hypomethylation is reported to play a crucial role in genomic instability and, consequently, carcinogenic processes [31, 32]. However, our results could not show any correlation between *LINE-1* methylation and *MLH1* or *DNMT1* expression, respectively. Previously, it was demonstrated in bladder cancer cells that *LINE-1* methylation was significantly decreased after treatment with H_2_O_2_ and reestablished after pretreatment with tocopherol acetate [64]. Similar effects could also be observed in our study under hyperglycemic conditions, where H_2_O_2_ tended to reduce *LINE-1* methylation provoking chromosomal instability. Though exclusive treatments with vitamin E could not reveal significant alterations, the combination of 25 *μ*M or 500 *μ*M H_2_O_2_ with 10 *μ*M vitamin E resulted in a significant increase in global methylation as compared to H_2_O_2_ treatment control. In line with our results on reduction of H_2_O_2_-induced MDA, these findings underline the exciting beneficial effects of the lower vitamin E concentration in counteracting oxidative stress, while acting cancer protective. However, whether *LINE-1* hypomethylation is a cause or effect of oxidative stress is surely a worthwhile focus for future research. Additional studies with other immortalized but also primary cell lines treated with further natural substances bearing antioxidative potential such as (−)-epigallocatechin gallate (Pointner et al. 2017, submitted for publication) are of great interest to assess cell line and substance-specific characteristics.

Taken together, we could demonstrate that vitamin E reduced H_2_O_2_-induced lipid peroxidation in a dose-dependent manner as well as caused lower increase of the DNA repair gene *MLH1*. Furthermore, *DNMT1* expression and global methylation were positively affected, all of them underlining the exciting beneficial effects of the lower concentration of vitamin E in counteracting oxidative stress. Moreover, our study revealed an influence by glucose concentration on MDA and ROS level as well as *DNMT1* expression, which is suggested to be linked to metabolic pathways. Thereby neither NAC nor the NOX inhibitor was able to alter all investigated parameters in the same way as vitamin E did. However, the assumed ROS induction and scavenging effect through one-time treatment was very likely to act in the short term. Furthermore, the highly reactive exogenous redox active compounds were neutralized after 48 h.

## 5. Conclusions

Antioxidative processes clearly affect main epigenetic enzymes regulation and presumably chromatin modification. Different impacts of glucose concentration indicate that physiological glucose levels need to be respected when analyzing interactions between antioxidative mechanisms and epigenetics. Vitamin E, especially in low concentrations, showed beneficial effects *in vitro* concerning oxidative stress as well as epigenetic alterations, revealing its cancer protective potential. Supplementation of exogenous antioxidants like vitamin E constitutes an effective means to counteract hyperglycemia-induced oxidative damage. Therefore, it bears a great potential for treatment and might even be used as possible approach in prevention of diseases such as obesity and diabetes.

## Figures and Tables

**Table 1:** Primer sequences used for MS-HRM analysis. *LINE-1*-rv contains an additional 5′ biotin residue for possible pyrosequencing use. Primer sequences were taken from literature as indicated. Amplicon length and CpG content were determined using BiSearch version 2.53 [65] and Ensembl release 86 database [66].

Primer	Sequence (5′-3′) for MS-HRM	Reference	Amplicon (bp)	CpGs
*MLH1*-fw	TTTTTTTAGGAGTGAAGGAGG	[67]	123	13
*MLH1*-rv	AACRCCACTACRAAACTAAA			
*DNMT1*-fw	GGTATCGTGTTTATTTTTTAGTAA	[68]	115	6
*DNMT1*-rv	ACGAAACCAACCATACCCAA			
*LINE-1*-fw	TTTTGAGTTAGGTGTGGGATATA	[69]	~143–148	~1–10
*LINE-1*-rv	AAAATCAAAAAATTCCCTTTC			

**Table 2:** Shared 48 h treatments by both glucose conditions per parameter.

	MDA	ROS	Superoxide	Expression		Methylation	
				*MLH1*	*DNMT1*	*MLH1*	*LINE-1*
Control	+	+	+	+	+	+	+
25 *μ*M H_2_O_2_				+	+	+	+
50 *μ*M H_2_O_2_				+	+	+	+
250 *μ*M H_2_O_2_	+	+	+				
500 *μ*M H_2_O_2_	+	+	+				
1 mM NAC	+	+	+				
250 *μ*M H_2_O_2_ + 1 mM NAC	+	+	+				
2 *μ*M VAS2870	+	+	+				
250 *μ*M H_2_O_2_ + 2 *μ*M VAS2870	+	+	+				
10 *μ*M vitamin E	+	+	+	+	+	+	+
50 *μ*M vitamin E	+	+	+	+	+	+	+
25 *μ*M H_2_O_2_ + 10 *μ*M vitamin E				+	+	+	+
25 *μ*M H_2_O_2_ + 50 *μ*M vitamin E				+	+	+	+
50 *μ*M H_2_O_2_ + 10 *μ*M vitamin E				+	+	+	+
50 *μ*M H_2_O_2_ + 50 *μ*M vitamin E				+	+	+	+
250 *μ*M H_2_O_2_ + 10 *μ*M vitamin E	+	+	+				
250 *μ*M H_2_O_2_ + 50 *μ*M vitamin E	+	+	+				
500 *μ*M H_2_O_2_ + 10 *μ*M vitamin E	+	+	+				
500 *μ*M H_2_O_2_ + 50 *μ*M vitamin E	+						

## Abbreviations

BER: Base excision repair
CIMP: CpG island methylation phenotype
CpG: Cytosine-guanine sequence
DNMT: DNA methyltransferase
LINE-1: Long interspersed nuclear element-1
M1dG: MDA deoxyguanosine adduct
MDA: Malondialdehyde
MLH1: MutL homolog 1
MMR: Mismatch repair
MS-HRM: Methylation-sensitive high-resolution melting
NAC: *N*-Acetylcysteine
NAD(P)H: Reduced form of nicotinamide adenine dinucleotide (phosphate)
NER: Nucleotide excision repair
NOX: NADPH oxidases
ROS: Reactive oxygen species.
